# A9 GLYCOCAGING IMPROVES THE THERAPEUTIC INDEX OF IBD DRUGS

**DOI:** 10.1093/jcag/gwaf042.009

**Published:** 2026-02-13

**Authors:** W Ma, B Cai, S C Menzies, J Kothandapani, C Wang, H Brumer, L M Sly

**Affiliations:** Pediatrics, The University of British Columbia Faculty of Medicine, Vancouver, BC, Canada; Pediatrics, The University of British Columbia Faculty of Medicine, Vancouver, BC, Canada; Pediatrics, The University of British Columbia Faculty of Medicine, Vancouver, BC, Canada; The University of British Columbia Michael Smith Laboratories, Vancouver, BC, Canada; The University of British Columbia Michael Smith Laboratories, Vancouver, BC, Canada; The University of British Columbia Michael Smith Laboratories, Vancouver, BC, Canada; Pediatrics, The University of British Columbia Faculty of Medicine, Vancouver, BC, Canada

## Abstract

**Background:**

Inflammatory bowel disease (IBD), encompassing Crohn’s disease (CD) and ulcerative colitis (UC), is marked by chronic inflammation along the gastrointestinal (GI) tract. Current therapies include oral small molecule drugs, such as corticosteroids and other immunomodulators. However, their use is limited by high doses required for efficacy due to premature systemic uptake, resulting in off-target effects. Notably, 30-40% of people with moderate to severe IBD do not respond to prednisone, the first-line corticosteroid for inducing remission. Although dexamethasone is more potent than prednisone or its active form, prednisolone, it is not used clinically due to systemic toxicity. These challenges highlight the need for more effective and safer treatments.

GlycoCage technology aims to improve delivery of IBD drugs. Drugs are linked to a plant carbohydrate and released only by gut bacterial glycosidases at the inflamed regions of the lower GI tract. We hypothesize that GlycoCaging will enhance therapeutic efficacy and reduce systemic side effects of corticosteroids.

**Aims:**

1) Compare the efficacy of free and caged prednisolone in two murine models of IBD. 2) Evaluate caged dexamethasone in two murine models of IBD. 3) Investigate systemic effects of free and caged corticosteroids.

**Methods:**

Two murine models were used to evaluate the GlycoCage system. In the SHIP-deficient (SHIP-/-) model of CD-like ileitis, dose titration started at 3 mg/kg for dexamethasone, 10 mg/kg for prednisone/prednisolone, or molar equivalent of their caged counterparts. Drugs were administered daily by oral gavage for 2 weeks. In the T cell transfer model of colitis, naïve CD45.1+ T cells were isolated by FACS and injected intraperitoneally into Rag-/- recipients. The same drugs were gavaged for 5 weeks to compare minimum effective doses. Treatment efficacy was assessed using histology, flow cytometry, and multiplexed cytokine assays. Systemic exposure was evaluated by measuring serum drug concentrations post-gavage using LC-MS/MS. Lung pathology in SHIP-/- mice was examined for off-target effects.

**Results:**

Responses to prednisone/prednisolone were variable, with prednisone showing lower efficacy than prednisolone. Caged prednisolone was as effective as the free drug. In contrast, dexamethasone eliminated intestinal inflammation in both models, and caged dexamethasone demonstrated a 10-fold reduction in minimum effective dose. Importantly, GlycoCaging limited drug concentrations in circulation and eliminated off-target effects in lungs observed with free drug.

**Conclusions:**

Our results highlight a novel prodrug strategy to broaden treatment options for IBD. Current findings posit GlycoCaged dexamethasone as a “best-in-class” steroid for IBD with greater efficacy and reduced off-target effects. Future work will evaluate additional GlycoCaged small molecule in preclinical models.

**Funding Agencies:**

CAG, CCC, CIHR

